# Knockdown of death receptor 5 antisense long noncoding RNA and cisplatin treatment modulate similar macromolecular and metabolic changes in HeLa cells

**DOI:** 10.55730/1300-0152.2634

**Published:** 2022-12-05

**Authors:** Dilek Cansu GÜRER, İpek ERDOĞAN VATANSEVER, Çağatay CEYLAN, Bünyamin AKGÜL

**Affiliations:** 1 Noncoding RNA Laboratory, Department of Molecular Biology and Genetics, Faculty of Science, İzmir Institute of Technology, İzmir, Turkey; 2 Department of Food Engineering, Faculty of Engineering, İzmir Institute of Technology, İzmir, Turkey

**Keywords:** HeLa cells, DR5-AS, cisplatin, FTIR spectroscopy, cancer, metabolism, transcriptomics

## Abstract

**Background/aim:**

Despite great progress in complex gene regulatory mechanisms in the dynamic tumor microenvironment, the potential contribution of long noncoding RNAs (lncRNAs) to cancer cell metabolism is poorly understood. Death receptor 5 antisense (DR5-AS) is a cisplatin inducible lncRNA whose knockdown modulates cell morphology. However, its effect on cell metabolism is unknown. The aim of this study is to examine metabolic changes modulated by cisplatin and DR5-AS lncRNA in HeLa cells.

**Materials and methods:**

We used cisplatin as a universal cancer therapeutic drug to modulate metabolic changes in HeLa cervix cancer cells. We then examined the extent of metabolic changes by Fourier transform infrared spectroscopy (FTIR). We also performed transcriptomics analyses by generating new RNA-seq data with total RNAs isolated from cisplatin-treated HeLa cells. Then, we compared cisplatin-mediated transcriptomics and macromolecular changes with those mediated by DR5-AS knockdown.

**Results:**

Cisplatin treatment caused changes in the unsaturated fatty acid and lipid-to-protein ratios and the glycogen content. These observations in altered cellular metabolism were supported by transcriptomics analyses. FTIR spectroscopy analyses have revealed that DR5-AS knockdown causes a 20.9% elevation in the lipid/protein ratio and a 76.6% decrease in lipid peroxidation. Furthermore, we detected a 3.42% increase in the chain length of the aliphatic lipids, a higher content of RNA, and a lower amount of glycogen indicating relatively lower metabolic activity in the DR5-AS knockdown HeLa cells. Interestingly, we observed a similar gene expression pattern under cisplatin treatment and DR5-AS knockdown HeLa cells.

**Conclusion:**

These results suggest that DR5-AS lncRNA appears to account for a fraction of cisplatin-mediated macromolecular and metabolic changes in HeLa cervix cancer cells.

## 1. Introduction

lncRNAs are transcripts of over 200 nucleotides (nt) in length that do not harbor any open reading frames (ORF) greater than 100 amino acids (aa) (Yao et al., 2019). lncRNAs are produced from quite versatile genomic loci following a complex biogenesis process in eukaryotic cells (Cao et al., 2018). As transcriptional and posttranscriptional regulators of gene expression, lncRNAs are involved in numerous cellular processes in health and disease, including cancer (DiStefano, 2018). lncRNAs function through one of four ways, including molecular signals, scaffolds, decoys and guides (Qian et al., 2020). Any deregulation in these molecular functions results in activation or suppression of numerous cancer-related signalling pathways (Erdogan et al., 2022). For example, HOTAIR promotes the cervical cancer progression by sponging miR-143-3p, which leads to enhanced proliferation and suppressed apoptosis through modulation of BCL2 expression (Liu et al., 2018). H19, which is highly expressed in cervical cell lines, has been reported to promote both anchorage-dependent and –independent growth without affecting apoptosis (Iempridee 2017). MALAT1 expression correlates well with the tumor size while its knockdown in cervical cancer cells leads to reduced proliferation and enhanced apoptosis (Yang et al., 2015). Additionally, many other lncRNAs have been shown to contribute to cervix cancer progression (Cáceres-Durán et al., 2020). Accumulating evidence clearly shows that numerous protein-coding genes play an instrumental role in orchestrating cancer cell metabolism under adverse conditions such as drug treatment (Martínez-Reyes and Chandel, 2021). Recently, emerging studies point to the vital role of lncRNAs in this process (Ghafouri-Fard et al., 2020).

We have recently reported that cisplatin, a universal chemotherapeutic drug (Rosenberg et al., 1965), induces a number of lncRNAs in HeLa cells (Gurer et al., 2021). Knockdown of death receptor 5 antisense (DR5-AS) lncRNA, one of those cisplatin-inducible lncRNAs, causes a morphological change in HeLa cells accompanied with a reduction in cell proliferation and metastasis. Despite a genomic overlap between DR5-AS and DR5, the knockdown or ectopic overexpression of DR5-AS is not sufficient to modulate the DR5 mRNA abundance or TRAIL-mediated apoptosis. Based on these transcriptomics and cellular data, DR5-AS has been proposed to account for some of the effects of cisplatin in HeLa cells (Gurer et al., 2021). However, it is unknown whether DR5-AS modulates cancer cell metabolism, similar to cisplatin, possibly linked to the proliferative or metastatic state of HeLa cells.

Fourier transform infrared spectroscopy (FTIR) is a noninvasive and rapid spectroscopic method for sensitive analysis of changes in biological systems at the functional group level. The spectra obtained can be processed through several digital manipulations to yield both qualitative and quantitative information based on the changes in bandwidths, intensities and band positions (Dogan et al., 2007; Cakmak et al., 2007). FTIR has several advantages such as, simultaneous measurement of spectral elements, a greater signal-to-noise ratio, and a greater optical throughput (Nguyen, 1985). Limitations of FTIR include difficulty in interpretation of interferograms, the spread of a local noise throughout the spectrum and the use of a single beam. FTIR is commonly used to measure changes in the concentration and composition of macromolecules, which provide valuable insight into disease- and drug-specific alterations (Manoharan et al., 1993; Ci et al., 1999) such as cancer metabolism or drug resistance (Wu et al., 2015).

In this study, we first used cisplatin as a universal chemotherapeutic drug to examine the extent of cisplatin-mediated metabolic changes in HeLa cervix cancer cells. FTIR analyses showed that cisplatin causes major metabolic changes in the unsaturated fatty acid ratio, lipid-to-protein ratio, glycogen and RNA contents. Transcriptomics analyses of cisplatin-treated cells supported these observations as revealed by deregulation of 1367 metabolic genes. Interestingly, knockdown of DR5-AS lncRNA in HeLa cells caused similar changes in the macromolecule patterns as revealed by FTIR spectroscopy analysis and perturbed the expression of similar metabolic genes albeit to a lesser extent. Bioinformatics analyses suggest that both cisplatin- and DR5-AS-knockdown-mediated changes appear to be orchestrated primarily by the PI3K signaling.

## 2. Materials and methods

### 2.1. Cell culture, transfection and cisplatin treatment in HeLa cells

Cell culture, transfection and cisplatin treatments were performed essentially as described previously (Gurer et al., 2021). Transfected cells were also subjected to crystal violet staining. Prior to cell imaging, transfected cells were fixed with cold methanol (100%) followed by staining with 0.1% crystal violet (Sigma, United States). Cells were incubated in dark at room temperature for five min. The excess dye was washed off under running tap water. Then the samples were air-dried and visualized under a brightfield microscope. To quantitatively measure the rate of apoptosis, three biological replicates were first trypsinized by 1X Trypsin-EDTA (Gibco, United States) and washed in 1X cold PBS (Gibco, United States). The cells were then resuspended in 1X Annexin binding buffer (Becton Dickinson, United States) and stained with Annexin V-PE (Becton Dickinson, United States) and 7AAD (Becton Dickinson, United States) prior to incubation for 15 min in dark at room temperature. The stained cells were analyzed by FACSCanto (Becton Dickinson, United States). Annexin V-positive cells were considered early apoptotic. A fraction of cells was stored at −80 °C for FTIR until use, while the rest was used for RNA isolation.

### 2.2. Total RNA isolation, RNA-seq and qPCR

Manufacturer’s instructions were followed to isolate total RNAs from control DMSO- and cisplatin-treated HeLa cells, using TRIzol (Life Technologies, United States). TURBO DNA-free^TM^ kit (Invitrogen, United States) was used to eliminate trace amounts of genomic DNA contamination from RNA samples.

RNA sequencing and bioinformatics analyses of control- and cisplatin-treated cells were performed essentially as described previously (Gurer et al., 2021). Briefly, libraries were prepared from five μg total RNAs by the poly(A) capture method (n = 3) and were sequenced by an Illumina HiSeq2500 (FASTERIS, Switzerland, https://www.fasteris.com/dna/). RNA-seq data obtained from cisplatin-treated HeLa cells were deposited into the Gene Expression Omnibus under the accession number GSE193447. RNA-seq data obtained from DR5-AS knockdown HeLa cells were reported previously under the accession number GSE160227 (Gurer et al., 2021). The following tools and the command lines were used in the conda environment (Grüning et al., 2018) to analyze RNA-seq data: FastQC (*fastqc -o <output_directory> – noextract <filename>*) (Andrews 2010); Trim Galore! (*trim_galore --quality 20 --fastqc --length 25 --output_dir* <output_directory> <input_file>) (Krueger 2015); SortMeRNA (*sortmerna –ref <reference_rRNA_sequence> --reads <trimmed_fastqc_file> --aligned <output_for_aligned_reads> --other <filtered_reads> --fastx –threads 10 –v*) (Kopylova et al., 2012); STAR (*STAR –genomeDir <index_path> --readFilesIn <trimmed&filtered_reads> --outFileNamePrefix <sequence_name> --outSAMtype BAM SortedByCoordinate –quantMode GeneCounts*) (Dobin et al., 2013); featureCounts (*featureCounts -O -a <annotation_file> -o <output_path/final_counts.txt> -g ‘gene_id’ -T 8 <list_of_BAM_files>*) (Liao et al., 2014); and MultiQC (by using *multiqc <results_directory> --outdir <output_directory>*) (Ewels et al., 2016). Differential gene expression analyses were performed by DESeq2 and pheatmap package in R (Durinck et al., 2009; Love et al., 2014). DESeq2 analyses were carried out with default parameters (Wald test). The results function was used with FDR for pAdjustMethod with a cutoff value of 0.05. Subsequently, significant results were filtered with a treshold of padj < 0.05. Finally, gene names, biotypes and gene descriptions were annotated via a custom code in R by using the dplyr package. Pathway Enrichment Analysis was performed with the Reactome database to identify biological processes associated with differentially expressed genes (Jassal et al., 2020). The resulting data was visualized with ggplot2 package in R platform (Wickham 2006). qPCR reactions were carried out as described previously (Gurer et al., 2021). GAPDH was used for normalization. qPCR primer sequences are listed in Table.

### 2.3. Fourier transform infrared spectroscopy (FTIR) measurements

Control, treated and transfected HeLa cells were lyophilized in a freeze drier (Labconco, FreeZone 18 L freeze dry system) overnight to remove water. The FTIR spectra of the samples were recorded in the 4000 and 450 cm^−1^ region for 20 scans at 4 cm^−1^ resolution using a Perkin Elmer spectrometer equipped with MIR TGS detector (Spectrum 100 Instrument, PerkinElmer Inc., Norwalk, CT, USA). Normalization (e.g., PO_2_ symmetric streching band) was performed according to a previously published procedure (Baran et al. 2013). The spectral data were analyzed using the Spectrum 100 software (PerkinElmer). The automatically baseline-subtracted spectra were then averaged for both control versus knockdown or treatment groups (n = 3). The spectra were normalized after baselining from two arbitrarily selected points for each analyzed region for the visual demonstration and comparison.

### 2.4. Statistical tests

The results are expressed as mean +/− standard deviation. The control and DR5-AS knockdown or treated Hela cells were statistically compared with the Mann–Whitney U test. The degree of significance was denoted as p < 0.05. For qPCR analyses of DMSO versus cisplatin treatments and negative GapmeR-versus DR5-AS GapmeR-transfected HeLa cells, unpaired t-test were conducted with a confidence level of 95%, p < 0.05.

## 3. Results

### 3.1. FTIR identifies macromolecular and metabolic changes in cisplatin-treated HeLa cells

**Figure 1:**
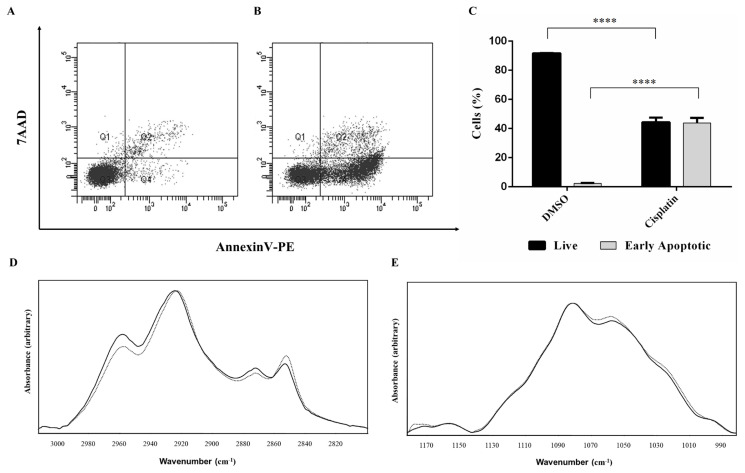
Cisplatin-induced apoptosis and metabolic changes in HeLa cells. 1X10^6^ HeLa cells were treated with 80 μM cisplatin (CP) and control 0.1% (v/v) DMSO for 16 h and analyzed by flow cytometry following staining with Annexin V-PE and 7AAD. Dot blots of (A) DMSO control and (B) cisplatin-treated cells were used to calculate the percentage of live and early apoptotic populations in (C). Q3 and Q4 quadrants depict live and early apoptotic cells, respectively. (D–E) FTIR spectra of the control (0.1% DMSO, solid line) and cisplatin-treated group (dotted line), respectively. The spectra were normalized with respect to the CH_2_ asymmetric mode (observed at 2924 cm^−1^) for the region between 3000–2800 cm^−1^ (D) and PO_2_^2−^ symmetric mode (observed at 1082 cm^−1^) for the region between 1181–894 cm^−1^ (E), respectively. ****: p ≤ 0.0001.

We used FTIR spectroscopy to inspect the cisplatin-mediated macromolecular and metabolic changes in HeLa cells (Dogan et al., 2013; Lopes-Rodrigues et al., 2017; de Magalhães et al., 2020). To this extent, we first treated HeLa cells with cisplatin as previously published (Gurer et al., 2021). Cisplatin at a concentration of 80 μM led to an early apoptotic rate of 43% (Figures 1A–1C). We then subjected three biological replicates of cisplatin-treated HeLa cells to FTIR analyses. The FTIR spectrum of HeLa cells exhibited several bands representing changes in different macromolecular functional groups. The region between 2800 cm^−1^ and 3700 cm^−1^ was used to analyze lipid and protein contents while the region between 890 cm^−1^ and 1478 cm^−1^ (the fingerprint region) was used for the analysis of nucleic acids, glycogen, lipids, and proteins.

2800–3012 cm^−1^ region: The FTIR spectrum in this region consists of CH_3_ and CH_2_ symmetric and asymmetric stretching vibrations. The band around 3006 cm^−1^ is an indicator of unsaturated fatty acids and denotes olefinic (C=C) bond stretching (Ozek et al., 2010) as shown in Figures 1D–1E. Since double bonds are oxidized via lipid peroxidation reactions, this band is also used as an indicator of the level of lipid peroxidation in cell and tissue culture-based studies (Ceylan et al., 2012). The intensity of this band decreased from 0.01283 (+/− 0.001773) to 0.011987 (+/− 0.000751) (p = 0.366) in cisplatin-treated HeLa cells, suggesting a 6.57% decrease in lipid peroxidation (Figures 1D–1E). Additionally, we used the intensity ratio of CH_2_ symmetric/CH_3_ symmetric stretching to estimate the lipid-to-protein ratio (Dogan et al., 2007) This ratio was 1.0769 (+/− 0.0515) and 1.3018 (+/− 0.0517) (p = 0.0012) in DMSO control and cisplatin-treated HeLa cells, respectively, pointing to a 20.9% increase in the lipid-to-protein ratio in cisplatin-treated HeLa cells (Figures 1D–1E). The intensity ratio of CH_2_ asymmetric/ CH_3_ asymmetric stretching was used as an indicator of hydrocarbon chain length of the lipid acyl chains (Gupta et al., 2014; Mylonis et al., 2019). This ratio was found to be 1.6910 (+/− 0.0164) for the cisplatin-treated HeLa cells and 1.48 (+/− 0.063) (p = 0.0012) for their control cells, suggesting a 14.39% increase in cisplatin-treated HeLa cells when compared to the control cells.

Fingerprint Region: The spectral region included several bands representing nucleic acids, glycogen, lipids, and proteins. The most striking cisplatin-mediated alterations within this region were centered between 1181 cm^−1^ and 890 cm^−1^ with peaks originating from basically nucleic acids and glycogen. Although the band at 1082 cm^−1^ depicting PO_2_ symmetric stretching of nucleic acids did not show any significant changes, the PO_2_ asymmetric stretching of nucleic acids band shifted from 1234.46 cm^−1^ (+/− 1.88) to 1235.73 cm^−1^ (+/− 0.73) (p = 0.18) in cisplatin-treated HeLa cells (Figures 1D–1E).

Attributes to cellular glycogen contents may be obtained by changes in the intensity of the band at 996 cm^−1^ (Wiercigroch et al., 2017). This band displayed a 7.03% decrease in cisplatin-treated HeLa cells. The other glycogen band characteristic of cancer cells (Wong et al., 1991) is around 1156 cm^−1^. We observed a decrease in the intensity of this band indicating reduction in the glycogen content of cisplatin-treated HeLa cells (Figures 1D–1E). The band at 1171 cm^−1^ is ascribed to C-O stretching of proteins in cancer cells (Rigas et al., 1990) and CO-O-C stretching of cholesterol. The intensity of this band increased by 31.29% in cisplatin-treated HeLa cells. The absorption around 1121 cm^−1^ belongs to RNA. Based on this spectrum, we observed 2.69% higher RNA content in cisplatin-treated HeLa cells when the band was normalized against the PO_2_ symmetric stretching band at 1083 cm^−1^ (Figures 1D–1E).

### 3.2. Cisplatin treatment perturbs the abundance of metabolic genes

**Figure 2:**
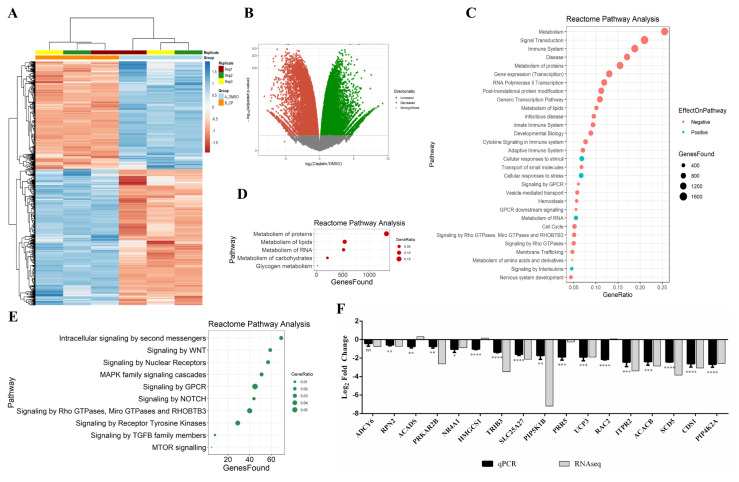
Transcriptomics analyses of cisplatin-treated HeLa cells. Total RNAs were isolated from three biological replicates of HeLa cells treated with 80 μM cisplatin and 0.1% DMSO as explained in Figure 1. The sequencing library was generated by the poly(A) capturing method and then sequenced with HiSeq 2500. Raw sequences were processed with the following tools in conda environment; quality check by FastQC, adapter trimming by Trim Galore!, rRNA contamination removal by SortMeRNA, reference genome alignment by STAR and read counting by featureCounts. Then the count matrix was subjected to differential expression analysis by DESeq2 in R. Only statistically significant ones (p < 0.05) were used for further analyses. (A) Heatmap of top 1000 differentially expressed genes (DEGs) in DMSO control and cisplatin-treated HeLa cells. (B) Volcano-plot of RNA-seq data analysis. (C) Reactome pathway analysis of differentially expressed protein-coding genes in cisplatin-treated HeLa cells. First 30 pathways were taken into account based on the GeneRatio values. (D,E) Reactome pathway analyses of other metabolic pathways associated with DEGs in cisplatin-treated HeLa cells and signal transduction pathways associated with metabolism-related DEGs, respectively. (F) qPCR analyses of differentially expressed metabolism-related genes. 1000 ng of RNA were used for cDNA synthesis and GAPDH was used for normalization. ns: nonsignificant, p > 0.05, *: p ≤ 0.05, **: p ≤ 0.01, ***: p ≤ 0.001, ****: p ≤ 0.0001.

**Figure 3:**
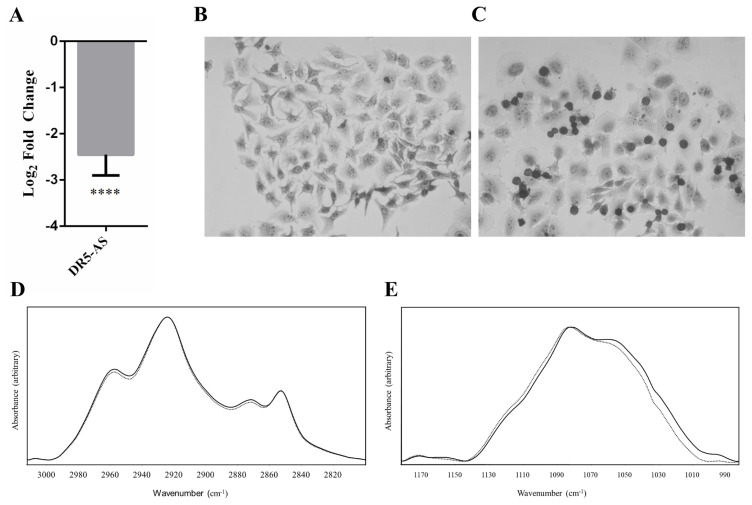
DR5-AS knocked-down cells have altered metabolism. (A) qPCR analyses were conducted with total RNAs isolated from negative and DR5-AS-GapmeR-transfected HeLa cells. 75,000 cells/well were employed for GapmeR-assisted transfection in a 6-well format. GapmeR concentration was 40 nM, and the cells were incubated for 72 h. Experiments were conducted in triplicates. (B, C) Brightfield images of negative GapmeR- and DR5-AS GapmeR-transfected HeLa cells, respectively, after crystal violet staining. (D, E) FTIR spectra of the control (negative GapmeR-solid line) and DR5-AS GapmeR-transfected group (dotted line), respectively. The spectra were normalized with respect to the CH_2_ asymmetric mode (observed at 2924 cm^−1^) for the region between (D) 3000–2800 cm^−1^ and PO_2_^2−^ symmetric mode (observed at 1082 cm^−1^) (E) for the region between 1181–894 cm^−1^, respectively. ****: p ≤ 0.0001.

Although previous studies showed the potential effect of cisplatin on metabolic genes in some cancer cells (Shirmanova et al., 2017; Miyazawa et al., 2019), a complete and in-depth profile of these genes in HeLa cells is not available yet. We employed the RNA sequencing approach to gain insight into the molecular mechanism of cisplatin-induced macromolecular and metabolic changes detected in HeLa cells through FTIR analyses (Figure 1). To this extent, we subjected three biological replicates of cisplatin-treated HeLa cells to RNA sequencing. We first checked the accuracy of data normalization. The MA plot showed that the RNA-seq data was normalized accurately (Supplementary Figure 1A). Our analyses yielded the differential expression of 16,452 transcripts, 8490 and 7962 of which were up- and down-regulated, respectively (Figures 2A–2B; Supplementary Table 1). We then carried on our analyses with 11,428 protein coding genes using the Gene Ontology and the Reactome pathway analysis tools (Jassal et al., 2020) to identify the biological processes primarily affected by cisplatin treatment (Figure 2C). Interestingly, metabolism is situated on top of the list sorted by GeneRatio. (Figure 2C), denoting the potential significance of cellular metabolism in cisplatin’s therapeutic effects. Gene ontology analysis results are presented in Supplementary Table 2. We then used the gene list sorted by the Reactome pathway analysis tool to interrogate which metabolic pathways are mainly affected by cisplatin treatment. Our analyses showed perturbations in protein metabolism (1326 genes), lipid metabolism (534 genes), RNA metabolism (512 genes), carbohydrate metabolism (207 genes) and glycogen metabolism (18 genes) (Figure 3D). We then selected these metabolic genes and performed another Reactome pathway analysis to uncover the signal transduction pathways associated with these metabolic genes. Our analysis revealed that 74 out of 1367 metabolic genes are linked with intracellular signaling with secondary messengers, e.g., PI3K (Figure 2E). All of these genomics analyses were validated by qPCR through the selection of a set of genes. Strikingly, there was a high congruity between RNA-seq and qPCR results (Figure 2F).

### 3.3. FTIR identifies macromolecular and metabolic changes in DR5-AS knockdown cells

It is well-documented that many protein-coding genes participate in the regulation of cancer cell metabolism (Pavlova and Thompson 2016). However, the potential role of long noncoding RNAs is only being embraced recently (Ghafouri-Fard et al., 2020). We have previously reported that cisplatin modulates the abundance of numerous lncRNAs in HeLa cells (Gurer et al., 2021). Interestingly, DR5-AS, one of those cisplatin inducible lncRNAs, partially accounts for some of the pleiotropic effects of cisplatin in HeLa cells, such as cell morphology, proliferation, and metastasis (Gurer et al., 2021). We hypothesized that DR5-AS could also modulate some, if not all, of cisplatin-mediated macromolecular and metabolic changes in HeLa cells. To this extent, we first knocked down DR5-AS using the GapmeR technology (Gurer et al., 2021). The qPCR analyses of total RNAs isolated from control and DR5-AS knockdown cells showed that DR5-AS could be knocked down by 82% (Figure 3A). We then examined the hexamethyl pararosaniline chloride (crystal violet) staining pattern of DR5-AS knockdown HeLa cells as this basic dye with high affinity for acidic intense chromatin structures during cell division can bind and stain both DNA and proteins. The microscopic analyses of knockdown cells stained with crystal violet exhibited a dramatic change in cell morphology and an intense staining pattern of dividing cells (Figures 3B–3C), suggesting cell viability, yet disturbed proliferation and probably metabolic changes (Kadoo et al., 2018).

We then examined the FTIR spectra of DR5-AS knockdown HeLa cells to identify macromolecular changes in morphologically disturbed cells for comparison with cisplatin-treated HeLa cells. The band around 3006 cm^−1^ decreased from 0.03181 (+/− 0.039) to 0.00747 (+/− 0.000701) for DR5-AS knockdown HeLa cells, exhibiting a 76.6% decrease in lipid peroxidation in DR5-AS knockdown HeLa cells (Figures 3D–3E). The intensity ratio of CH_2_ symmetric/CH_3_ symmetric stretching, which is used to estimate the lipid/protein ratio (Dogan et al., 2007), increased to 1.3018 in DR5-AS knockdown HeLa cells compared to 1.076 in control HeLa cells indicating a 20.9% increase (p = 0.0173) (Figures 3D–3E). Additionally, the intensity ratio of CH_2_ asymmetric/CH_3_ asymmetric stretching was used as an indicator of the hydrocarbon chain length of the lipid acyl chains (Gupta et al., Mylonis et al., 2019). This ratio was found to be 1.630 (+/− 0.030) for the DR5-AS knockdown HeLa cells and 1.5359 (+/− 0.1186) (p = 0.0173) for their control cells. Based on this ratio, we calculated a 6.5% increase in the DR5-AS knockdown HeLa cells when compared to the control cells.

In the fingerprint region, the band at 1082 cm^−1^, which represents PO_2_ symmetric stretching of nucleic acids, shifted from 1080.92 (+/− 1.71) cm^−1^ to 1083.09 (+/− 0.33) cm^1^ (p = 0.026) in DR5-AS knockdown HeLa cells. In addition, the PO_2_ asymmetric stretching of nucleic acids band shifted from 1234.52 cm^−1^ (+/− 0.41) to 1235.47 (+/−0.45) (p = 0.0152) in DR5-AS knockdown HeLa cells (Figures 3D–3E). The band at 996 cm^−1^, which is attributed to the cellular glycogen content (Wiercigroch et al., 2017), almost vanished in DR5-AS knockdown HeLa cells. The other glycogen band characteristic of cancer cells (Wong et al., 1991) is present at 1156 cm^−1^, which also decreased in intensity indicating a decrease in the glycogen content of DR5-AS knockdown cells. The band at 1171 cm^−1^ is ascribed to C-O stretching of proteins in cancer cells (Rigas et al., 1990) and CO-O-C stretching of cholesterol. The intensity of this band increased by 9.66% in the DR5-AS knockdown HeLa cells.

One of the most important changes in the spectra is the subtle increase in the RNA content of DR5-AS knockdown cells as depicted by the intensity of the band at 1121 cm^−1^ (Ozek et al., 2010). Our calculations indicate a 16.68% higher RNA content in DR5-AS knockdown HeLa cells when the band was normalized with respect to the PO_2_ symmetric stretching band at 1083 cm^−1^ (Figures 3D–3E).

### 3.4. Expression patterns of metabolic genes are similar in DR5-AS knockdown and cisplatin-treated HeLa cells

**Figure 4:**
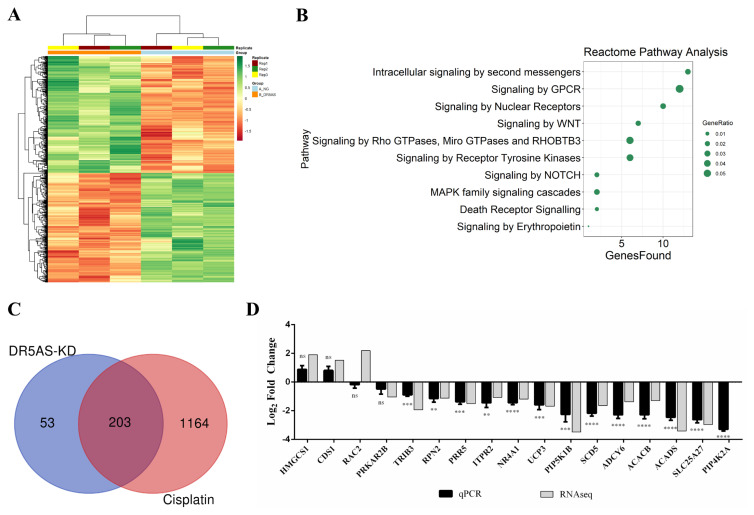
Common metabolic gene expression pattern in cisplatin-treated and DR5-AS knockdown HeLa cells. The GSE160227 RNA-seq data of DR5-AS knockdown cells were reanalyzed to have count matrix with featureCounts in conda environment. Count matrix was further processed by DESeq2 and (A) Heatmap of top 1000 DEGs was generated with pheatmap package in R. (B) Reactome pathway analysis of signal transduction pathways in DR5-AS knockdown cells was performed with genes associated with metabolic pathways. (C) Venn diagram of metabolism-related DEGs in DR5-AS knockdown and cisplatin-treated HeLa cells. (D) qPCR analyses of metabolism-related genes differentially expressed in DR5-AS knockdown HeLa cells. 1000 ng RNA was used for cDNA synthesis and GAPDH was used for normalization. ns: nonsignificant, p > 0.05, *: p ≤ 0.05, **: p ≤ 0.01, ***: p ≤ 0.001, ****: p ≤ 0.0001.

The FTIR spectra of DR5-AS knockdown and cisplatin-treated HeLa cells suggest that at least a fraction of cisplatin-mediated metabolic changes could be accounted for by DR5-AS. To solidify these observations, we reexamined the previously published RNA-seq data obtained from DR5-AS knockdown cells (Gurer et al., 2021). Our analyses revealed the differential expression of 256 genes (51 upregulated, 152 downregulated) associated with metabolism in DR5-AS knockdown HeLa cells (Figure 4A). When we fed these metabolic genes into the Reactome pathway analysis tool, we discovered the intracellular signaling by second messengers to be on top of the list, parallel to our findings in cisplatin-treated HeLa cells (Figure 4B). Interestingly, DR5-AS knockdown modulated the abundance of 185 out of 256 metabolic genes parallel to cisplatin treatment (40 upregulated, 145 downregulated) (Figure 4C; Supplementary Table 3). Pearson correlation analysis further supported their moderate positive correlation (Supplementary Figure 1B, r = 0.72, p < 0.05). We then selected some of the key metabolic genes for qPCR validation. As expectedly, qPCR results were highly congruous with the RNA-seq data (Figure 4D).

## 4. Discussion

A complex interaction among various types of cells in the tumor microenvironment (TME) contributes to the overall cancer cell metabolism (Lau and Vander Heiden 2020), which can be targeted for cancer chemotherapy by using several different chemical agents. Although cisplatin has been used in the treatment of various cancers for long, its effect on HeLa cell and other cancer cell metabolism is partially investigated. Recently, FTIR analyses have been successfully employed to investigate the molecular structure, reactivity, and cytotoxicity of cisplatin (Wang et al., 2015; Farhadi et al., 2016). A group from Iran studied cisplatin resistance on ovarian cancer cells (Zendehdel et al., 2012) by using FTIR spectroscopy. Their results displayed two main changes parallel with the resistant cancer cells: the increase in the β-sheets in protein secondary structures and a shift towards higher wavenumbers of CH_2_ bands which is ascribed to the condensation of the aliphatic chains in the fatty acids of phospholipids of the cancer cell membranes. In our study, we did not consider protein secondary structural changes which possibly occur due to endoplasmic reticulum stress which was shown to play an important role in cisplatin resistance in oral cancer cells (Chen et al., 2020). β-sheets are known to occur from α-helices in the protein aggregation studies as a result of ER stress and unfolded protein response (Hamdan et al., 2017). In the present study, we report the metabolic and macromolecular structural changes in cisplatin-treated and DR5-AS knockdown HeLa cells. We further show for the first time that DR5-AS lncRNA accounts for some of the cisplatin-mediated metabolic and macromolecular changes in HeLa cells by exploiting FTIR spectroscopy and transcriptome analyses.

Transcriptome analyses are also widely used to gain molecular insight into metabolic and cellular changes. For example, RNA-seq analyses of cisplatin and phenanthriplatin were conducted in A549 nonsmall cell lung cancer cells to uncover the most affected biological processes (Monroe et al., 2020). In this study, the authors primarily focused on apoptosis, cytoskeleton, cell migration, and proliferation. Although we observed a similar expression pattern in HeLa cells of several genes, such as ATF3, CDKN1A, GADD45A, and FDXR, our analyses highlighted metabolism as one of the most affected biological processes (Figure 2C). Another RNA-seq analysis was conducted on cisplatin-resistant HeLa cells to explore gene expression patterns associated with DNA methylation and drug resistance (Zeller et al., 2012). Both studies disregarded cisplatin-induced gene expression patterns associated with cancer cell metabolism.

Although it is evident that several key protein-coding genes play an instrumental role in orchestrating cancer cell metabolism (Martínez-Reyes and Chandel 2021), the vital role of lncRNAs in this process is emerging recently (Ghafouri-Fard et al., 2020). Both oncogenic and tumor suppressor lncRNAs might regulate cancer cell metabolism. For example, HOTAIR was reported to promote cancer cell energy metabolism in pancreatic adenocarcinoma by upregulating hexokinase-2 (Ma et al., 2019). NEF lncRNA, which is downregulated in NSCLC tissues, targets glucose transportations to modulate lung tumorigenesis (Chang et al., 2019). Recently, we reported changes in the abundance of numerous lncRNAs in cisplatin-treated HeLa cells and DR5-AS appears to account for some of the cisplatin-induced cellular phenotypes (Gurer et al., 2021). It is interesting that although DR5-AS is a cisplatin-inducible lncRNA (Gurer et al., 2021), its knockdown in the absence of cisplatin also generates a cisplatin-like phenotype (Figure 3B). This observation suggests that DR5-AS could be a prosurvival lncRNA expressed under stress conditions (e.g., cisplatin treatment). However, more studies are required to delineate the molecular function of DR5-AS. As a modulator of cell proliferation and metastasis, DR5-AS appears to modulate HeLa cell metabolism as evident by the differential expression of numerous metabolic genes upon DR5-AS knockdown (Figure 4A). Interestingly, a great fraction of these metabolic genes (Figure 5C, 185 out of 256 genes) is differentially expressed upon cisplatin-treatment as well, suggesting that DR5-AS could be used as a drug target to modulate HeLa cell metabolism. DR5-AS is expressed in Jurkat cells and is inducible by cisplatin to a greater extent compared to HeLa cells (Gurer et al., 2021). It would be interesting to examine whether DR5-AS is a general modulator of cancer cell metabolism or specific to HeLa cells.

Our transcriptome analyses correlated well with FTIR analyses. For example, we monitored a 6.75% and 76.6% decrease in the intensity of the band around 3006 cm^−1^ in cisplatin-treated and DR5-AS knockdown HeLa cells, respectively (Figures 1D–1E; Figures 3D–3E). As this band is an indicator of =CH stretching vibrations of lipids (Severcan et al., 2005) and potential lipid peroxidation events, we postulate that the decrease in the unsaturation of the lipids corresponds to an increase in the saturation status and antioxidant response of the cells. SLC25A27 (a.k.a Uncoupling Protein 4, UCP4), which, uncouples oxidation of substrates from ADP phosphorylation in mitochondria by modulating membrane potential, is a prognostic marker in breast cancer (Gonidi et al., 2011; Lytovchenko and Kunji 2017). Interestingly, we detected a 4.3-fold decrease in the expression of SLC25A27 in cisplatin-treated and DR5-AS knockdown HeLa cells (Supplementary Table 3) and a 3.2-fold decrease in the expression of UCP3 as the other important mitochondrial protein that plays important roles in the mitochondrial ROS metabolism. Metabolic changes in cisplatin-treated and DR5-AS knockdown HeLa cells also included an increase in the lipid/protein ratio (Figures 1D–1E; Figures 3D–3E). This could be attained by an increase in the total amount of lipids or a decrease in protein content or a combination of both. The decrease in the expression of ACADS transcription suggests a decrease in fatty acid oxidation as this enzyme is one of the earlier enzymes involved in this enzymatic cascade (Camões et al., 2015). The decrease in mitochondrial metabolism is further supported by the increase in the expression of SNAI1. Similarly, the decreases observed in the expression of ACACB and HMGCS1 genes as key players in the synthesis of two important members of neutral lipids indicate a potential reduction in the neutral lipid synthetic pathways. FTIR analyses were supported by gene expression analyses as the metabolism of proteins and lipids are the most affected metabolic activities upon cisplatin treatment (Figure 2D). Strikingly, the expression of several genes was deregulated, such as HSD3B7, CDS1, ACSL1, HMGCS1, OSBPL10, and CYP1A1, which are associated with lipid storage and cholesterol metabolism. In addition to the neutral lipid biosynthetic and catabolic pathways, both cisplatin treatment and DR5-AS knockdown suggest a decrease in the lipid unsaturation thought to be mediated by a decrease in the SCD5 expression. The decrease in lipid unsaturation could be a way to remedy the toxic effects of cisplatin treatment.

All of these observations are further supported by the finding that the majority of differentially expressed metabolic genes are primarily associated with the PI3K-Akt pathway, which is known to modulate cancer cell metabolism (Hoxhaj and Manning 2020). Our gene expression analyses suggest that cisplatin treatment suppresses the PI3K-Akt pathway (Figure 2), which is in agreement with previous studies (Zhang et al., 2016; Gohr et al., 2017; Lin et al., 2017). Suppression of the PI3K-Akt pathway could potentially decrease the amount of related enzymes such as FOXO via PI3K-akt-FOXO pathway. The PI3K/AKT/FOXO axis is also known to inhibit fatty oxidation within the cancer cell metabolism (Link and Fernandez-Marcos 2017). It would be interesting to examine whether reduced levels of ITPRs are associated with diminished levels of calcium release from ER and calcium uptake by mitochondria as such events might lead to lower levels of apoptosis and oxidative stress (Bánsághi et al., 2014). In fact, inhibition of the PI3K-Akt pathway is employed to sensitize cancer cells to treatment with cisplatin (Zhang et al., 2016; Gohr et al., 2017). To this extent, DR5-AS knockdown could be a potential avenue for cancer treatment by sensitizing cells to cisplatin-mediated cell death by inactivating the PI3K-Akt pathway.

## Supplementary Figure

Supplementary Figure 1 MA plot for read counts (A) shows RNAseq analysis of 80 uM cisplatin treated HeLa cells. X axis shows the normalized mean and Y axis indicates log2 fold change values. Red points correspond to significant genes (FDR, padj < 0.05) while grey points indicate non-significant ones. Positive area above middle line (light red) shows upregulated genes and the negative area have downregulated genes. Pearson’s Correlation scatterplot shows the relationship between CP and DR5-AS knockdown RNAseq datasets. Pearson’s r coefficient 0.72 indicates positive moderate correlation between two samples.

## Figures and Tables

**Table:** Primer sequences that were used in this study.

*Gene Name*	*Gene Symbol*	*Forward 5′-3′*	*Reverse 5′-3′*
*Uncoupling Protein 3*	UCP3	GGTCTCACCTCTAGACAACCG	GGACAATGCCTTGGGAGAGA
*Solute Carrier Family 25 Member 27*	SLC25A27	CAGCTACATACCACCGGCTC	TCCATAACCGCAGCCATCC
*CDP-Diacylglycerol Synthase 1*	CDS1	ACTCACCTCGCAAGAAGGAAG	GGAGCAAGGGGACAAAGATGA
*3-Hydroxy-3-Methylglutaryl-CoA Synthase 1*	HMGCS1	AATGCCCTGCCCCTATTCTT	GTTGCAGTCCTTCTTTGCACC
*Acyl-CoA Dehydrogenase Short Chain*	ACADS	GGCAGTTACACACCATCTACCA	CGGCATGTCTGGAGCAACA
*Acetyl-CoA Carboxylase Beta*	ACACB	TGGAGGGCCACTTGAGTTAC	CAGTCCAGTCCTCAGTCACC
*Stearoyl-CoA Desaturase 5*	SCD5	TCCCCCTTCTGTCTTCTACCC	TCAGTGTAGTTCAGGAGAGCATTT
*Proline Rich 5*	PRR5	CACCTGCCCACAGAGAATGTA	GTGCTGGAGTCTGGTCACTT
*Tribbles Pseudokinase 3*	TRIB3	CTGGCATCCTTGAGCTGACA	AGGCCGACACTGGTACAAAG
*Rac Family Small GTPase 2*	RAC2	GATGGTGTCCTTGTCGTCCC	CACCACTGCCCCAGCAC
*Nuclear Receptor Subfamily 4 Group A Member 1*	NR4A1	GGTGACCCCACGATTTGTCT	GGCTTATTTACAGCACGGCG
*Phosphatidylinositol-4-Phosphate 5-Kinase Type 1 Beta*	PIP5K1B	TTGCTGTTGCTTGCTGAACTG	AGCACTTCCAACTCCAAAAAGG
*Phosphatidylinositol-5-Phosphate 4-Kinase Type 2 Alpha*	PIP4K2A	CCTGAACCAGTACACACGGA	ACGCAGACACTCTCCCTAAC
*Adenylate Cyclase 6*	ADCY6	CAGACAGACAGGGAAGGGTAGG	GAGGGGTCAGTAGCAGAAGCA
*Inositol 1,4,5-Trisphosphate Receptor Type 2*	ITPR2	CAGCAGAAACTGATACTTTTGAACC	ATGCAATCATCTTGAACTTTGATGT
*Protein Kinase CAMP-Dependent Type II Regulatory Subunit Beta*	PRKAR2B	AGTTGCCCTGTTTGGAACGA	TGTCACTACAGGTCTTGCTCC
*Ribophorin II*	RPN2	TCCCCAACACACAGCAGATAC	AAGCAACATCTGGGTAAGTTAGGA
