# Supplementary figures and images for: Knockdown of death receptor 5 antisense long noncoding RNA and cisplatin treatment modulate similar macromolecular and metabolic changes in HeLa cells
Source: Turk J Biol. 2022 Dec 5;46(6):488–500. doi: 10.55730/1300-0152.2634 (PMC10387844; doi:10.55730/1300-0152.2634)

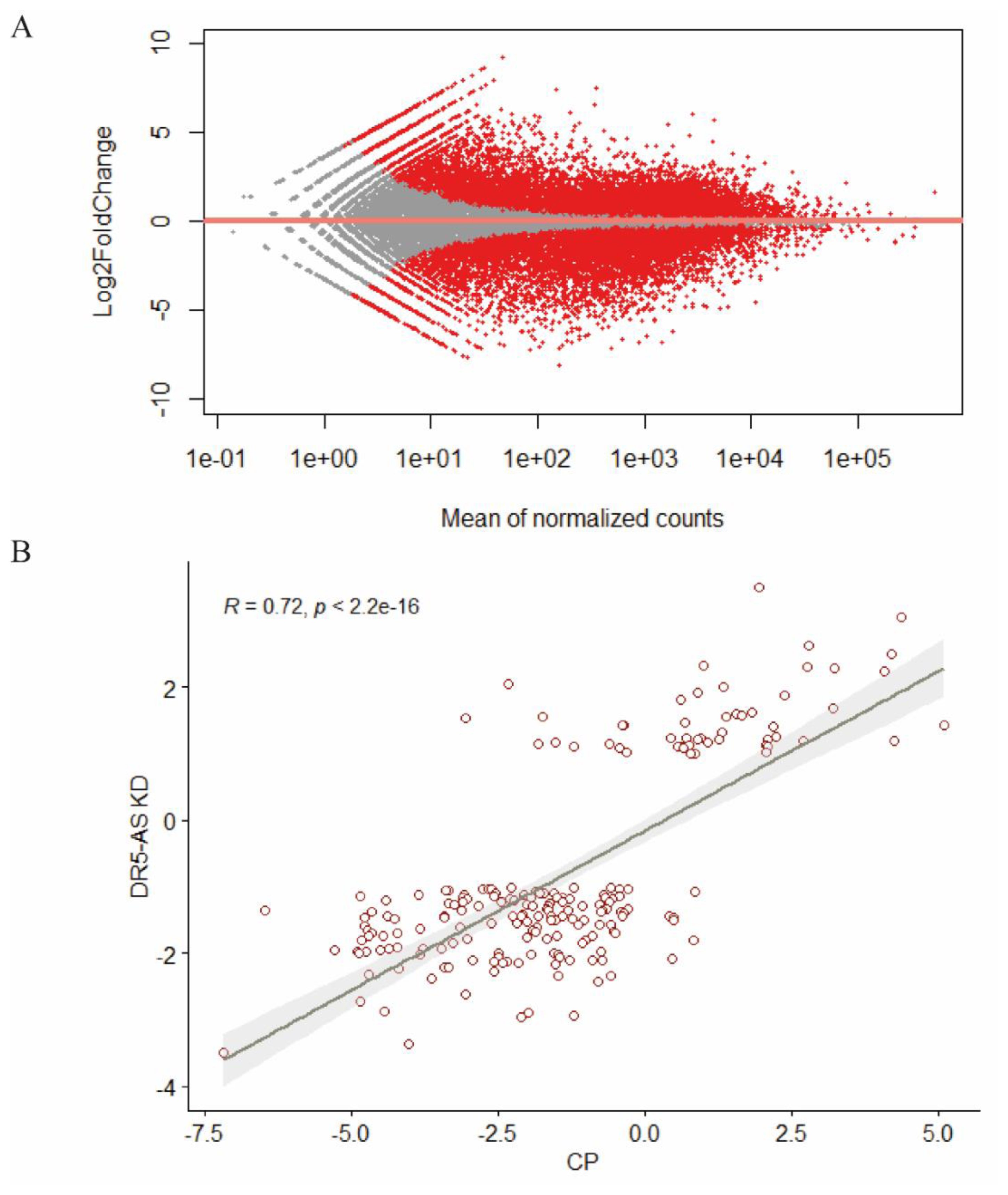

Supplement: Supplementary Figure 1 — MA plot for read counts (A) shows RNAseq analysis of 80 uM cisplatin treated HeLa cells. X axis shows the normalized mean and Y axis indicates log2 fold change values. Red points correspond to significant genes (FDR, padj < 0.05) while grey points indicate non-significant ones. Positive area above middle line (light red) shows upregulated genes and the negative area have downregulated genes. Pearson’s Correlation scatterplot shows the relationship between CP and DR5-AS knockdown RNAseq datasets. Pearson’s r coefficient 0.72 indicates positive moderate correlation between two samples. [file turkjbiol-46-6-488s1.tif]
